# Supplementary material for: A high-resolution mRNA expression time course of embryonic development in zebrafish
Source: eLife. 2017 Nov 16;6:e30860. doi: 10.7554/eLife.30860 (PMC5690287; doi:10.7554/eLife.30860)
Supplement: Supplementary file 6. [file elife-30860-supp6.zip › biolayout-clusters-files/Cluster245-genes.html]

Cluster245


# Cluster245: Genes

| Ensembl ID | Gene Name | Chr | Start | End | Biotype |
| --- | --- | --- | --- | --- | --- |
| ENSDARG00000035573 | AK6 | 5 | 25599384 | 25603092 | protein\_coding |
| ENSDARG00000040666 | nifk | 9 | 38348775 | 38352300 | protein\_coding |
| ENSDARG00000033370 | ppil3 | 5 | 64170822 | 64174869 | protein\_coding |
| ENSDARG00000013478 | rrp15 | 16 | 4367643 | 4373733 | protein\_coding |
| ENSDARG00000004452 | tardbpl | 23 | 29431249 | 29436304 | protein\_coding |
| ENSDARG00000103530 | zgc:113102 | 3 | 7377369 | 7570829 | protein\_coding |
